# Phenotype expansion of variants affecting p38 MAPK signaling in hypospadias patients

**DOI:** 10.1186/s13023-022-02334-5

**Published:** 2022-05-23

**Authors:** Defu Lin, Huakang Du, Sen Zhao, Bowen Liu, Hongcheng Song, Guannan Wang, Weiping Zhang, Haiyan Liang, Pei Liu, Chao Liu, Wenwen Han, Zhenwu Li, Yang Yang, Shuofan Chen, Lina Zhao, Xiaoxin Li, Zhihong Wu, DISCO (Deciphering Disorders Involving Scoliosis & COmorbidities) study group, Guixing Qiu, Zhihong Wu, Terry Jianguo Zhang, Nan Wu, Shengru Wang, Jiaqi Liu, Sen Liu, Yuzhi Zuo, Gang Liu, Chenxi Yu, Lian Liu, Jiashen Shao, Sen Zhao, Zihui Yan, Hengqiang Zhao, Yuchen Niu, Xiaoxin Li, Huizi Wang, Congcong Ma, Zefu Chen, Bowen Liu, Xi Cheng, Jiachen Lin, Huakang Du, Yaqi Li, Shuang Song, Weijie Tian, Zhixin Xie, Zhengye Zhao, Lina Zhao, Zhi Zhao, Zhifa Zheng, Yingzhao Huang, Ning Sun, Nan Wu

**Affiliations:** 1 Department of Urology, Beijing Children’s Hospital, Capital Medical University, National Center for Children’s Health, Beijing, 100045 People’s Republic of China; 2 Department of Orthopedic Surgery, State Key Laboratory of Complex Severe and Rare Diseases, Peking Union Medical College Hospital, Chinese Academy of Medical Sciences, Beijing, 100730 People’s Republic of China; 3 Beijing Key Laboratory for Genetic Research of Skeletal Deformity, Beijing, 100730 People’s Republic of China; 4 Key Laboratory of Big Data for Spinal Deformities, Peking Union Medical College Hospital, Chinese Academy of Medical Sciences, No. 1 Shuaifu Road, Beijing, 100730 People’s Republic of China; 5 Medical Research Center, Peking Union Medical College Hospital, Peking Union, Beijing, 100730 People’s Republic of China

**Keywords:** Hypospadias, Pedigree, Proto-oncogene proteins B-raf (BRAF), Mitogen-activated protein kinases (MAPK), p38 mitogen-activated protein kinases, Sex-determining region Y protein (SRY)

## Abstract

**Background:**

Hypospadias is a congenital anomaly of the male urogenital system. Genetics factors play an important role in its pathogenesis. To search for potential causal genes/variants for hypospadias, we performed exome sequencing in a pedigree with three patients across two generations and a cohort of 49 sporadic patients with hypospadias.

**Results:**

A novel *BRAF* variant (NM_004333.6: c.362C > A) was found to co-segregate with the hypospadias phenotype in the disease pedigree. In cells overexpressing the *BRAF* mutant, the phosphorylation level of p38 MAPK was significantly increased as compared with the cells overexpressing the wild-type *BRAF* or RASopathy-related *BRAF* mutant. This variant further led to a reduced transcription level of the *SRY* gene, which is essential for the normal development of the male reproductive system. In the cohort of sporadic patients, we identified two additional variants in p38 MAPK signaling-related genes (*TRIM67* and *DAB2IP*) potentially associated with hypospadias.

**Conclusion:**

Our study expands the phenotypic spectrum of variants affecting p38 MAPK signaling toward the involvement of hypospadias.

## Background

Hypospadias is a congenital anomaly of the male urogenital system with an incidence of 20.9 per 10,000 births [1]. It can be isolated, accompanied by other genitourinary abnormalities, or co-exist with multisystemic anomalies [2–4]. Genetic variant is a major etiologic factor of hypospadias. [2, 3, 5]. The heritability of hypospadias varies from 57 to 77% among different studies, suggesting the key role of genetic factors in the pathogenesis of hypospadias [2, 6].

The development of male external genitalia undergoes two phases: a hormone-independent phase and a hormone-dependent phase, both under complex genetic regulation [3–5]. In the hormone-independent phase, key genes involved in WNT/β‐catenin signaling (*WNT5A*), Sonic Hedgehog signaling (*SHH*, *GLI3*), fibroblast growth factor signaling (*FGF8*, *FGFR2*), and bone morphogenetic protein signaling (*BMP7*) pathways participate in the outgrowth of genital tubercle and the canalization of urethral plate. Single nucleotide polymorphisms or pathogenic variants in these genes have been associated with hypospadias [4, 5, 7, 8]. In the hormone-dependent phase driven by the increased androgen level, the lateral folds of the urethral groove fuse to form the urethra [8]. Genes that participate in testicular determination (*SRY*, *SOX9*, *WT1*, *NR5A1*, *MAP3K1*, etc.), androgen synthesis and metabolism (*SRD5A2*, *CYP11A1*, *POR*, *STAR*, *MAMLD1*, etc.), or androgen signaling (*AR*, *DGKK*, etc.) are key regulators of this process. Pathogenic variants in these genes can cause deficit androgen production or perturbation of androgen signaling, thereby causing hypospadias or complete feminization of the external genitalia [4, 5, 9, 10].

Although numerous causal genes related with hypospadias have been reported, the etiology remains unidentified in over 70% of hypospadias patients [5, 11]. To search for potential causal genes/variants for hypospadias, we performed exome sequencing in a Chinese pedigree with three hypospadias patients and identified a novel variant in the *BRAF* gene. The subsequent functional analysis revealed that this variant could lead to an increased p38 MAPK activation. To elucidate the contributions of the p38 MAPK signaling pathway to hypospadias, we further studied mutations in other genes involved in the p38 MAPK signaling pathway in a sporadic cohort of 49 hypospadias patients.

## Results

### Demographic and clinical characteristics

**Fig. 1:**
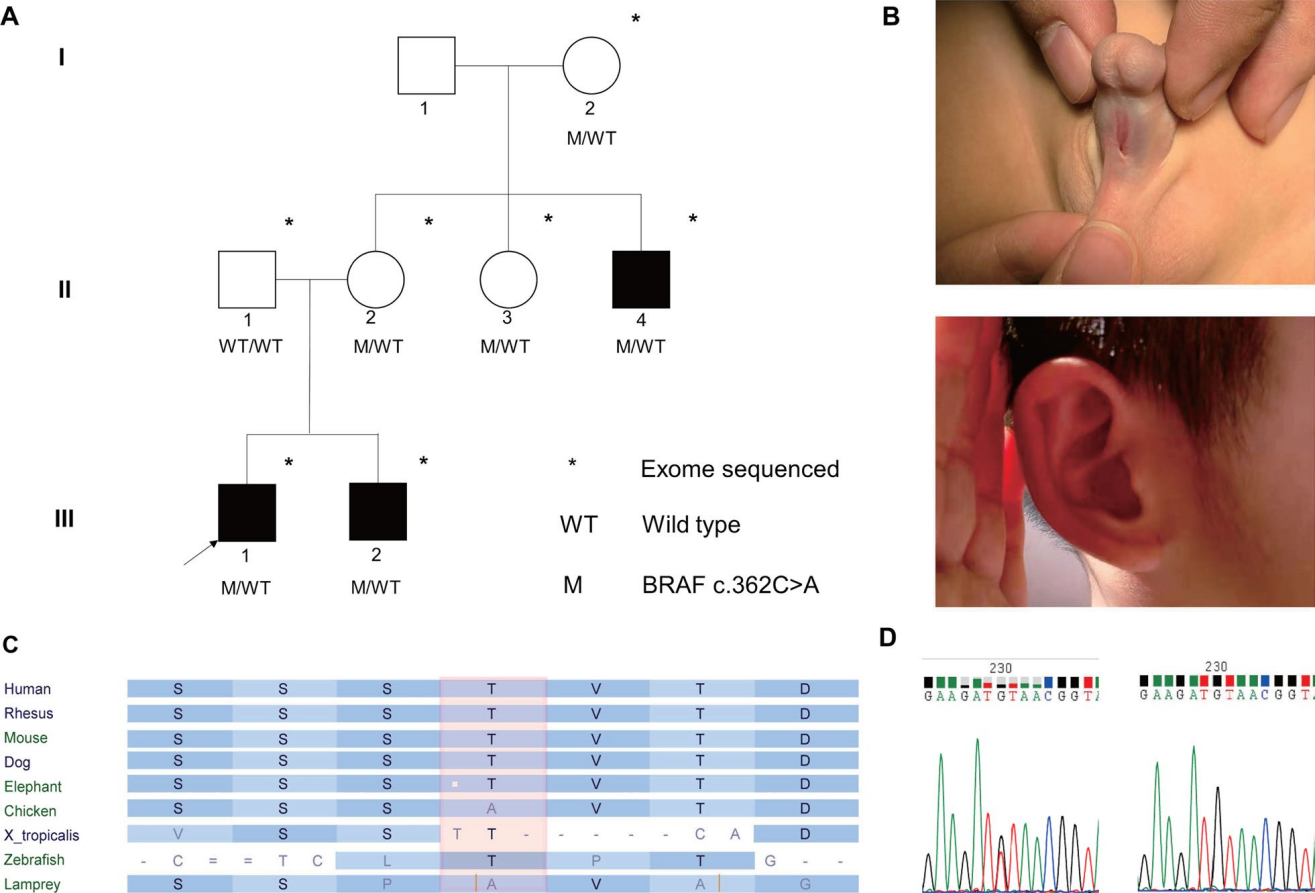
**A** Pedigree of the hypospadias patients. * indicates members who were exome-sequenced. M: the mutated allele *BRAF* c. 362C > A; WT: wildtype BRAF allele; **B** Clinical evaluation of the patients. Up: the proband has penoscrotal hypospadias. Down: III-2 has mild constricted ear (notice the folded helical rim); **C** Conservation of the variant loci; **D** Sanger sequencing confirmed the variant. Left: heterozygous mutated allele; Right: wildtype allele

We recruited a pedigree with three cases affected by proximal hypospadias (Fig. 1A, B) and a sporadic cohort with individuals affected by isolated hypospadias of various severity, ranging from penoscrotal to glandular hypospadias. None of the patients reported exposure to exogenous progesterone or potential teratogenic drugs during pregnancy. Testicular size and function are within the normal range among the patients (Table 1**)**.

**Table 1:** Demographic and clinical information of the patients

Participant	III-1	III-2	II-4
Hypospadias	Proximal penile shaft	Proximal penile shaft	Penoscrotal
Birth weight	3.3	3.4	1.75
Current age	6.5	3.5	35
Current weight (kg)	22 (45th percentile)	14(16th percentile)	70
Current height (kg)	120 (48th percentile)	100(49th percentile)	165
Congenital Heart Disease	NEG	NEG	NEG
Neurologic disorder	NEG	NEG	NEG
Cutaneous abnormality	NEG	Hyperpigmented patch on the back(~ 20 cm)	Partial curly hair
Craniofacial dysmorphism	NEG	Mild constricted ear	NEG

**“**NEG” indicates negative findings

### A novel *BRAF* variant co-segregating with hypospadias in the pedigree

We first performed a co-segregation analysis in the three-generation pedigree. Exome sequencing of the pedigree yielded a total of 2651 variants that passed the quality control (QC). We excluded the variants that are present in public databases, leaving 556 single-nucleotide variations and 34 insertion/deletion variants (Table 2). Among these variants, four variants were found to co-segregate with the disease, including *BRAF* c.362C > A, *FRAS1* c.7004 T > G, *ELF4* c.1185G > A, and *MESP2* c.829G > A (Table 3).

**Table 2:** Variant prioritization process

Filtration steps	SNVs	indels
QC-passed variants	2528	123
Absent from public databases	556	34
Protein-altering	555	34
Variants segregated with disease	4	0
Predicted deleterious	NM_004333.4(BRAF): c.362C > A(p.Thr121Lys)	

QC: quality control, SNVs: single nucleotide variants, indels: insertion-deletion mutations

**Table 3:** Detailed information of the candidate variants

Gene	Variant	Amino acid change	Variant effect	Associated disease	MAF	pLI	missense Z-score	GERP + +	CADD
*BRAF*	NM_004333.5:c.362C>A	p.Thr121Lys	Missense	RASopathies	0	1	3.72	5.56	22.7
*FRAS1*	NM_025074.6:c.7004T>G	p.Leu2335Arg	Missense	Fraser syndrome 1	0	0	0.08	5.49	14.28
*ELF4*	NM_001421.3:c.1185G>A	p.Val395 =	Synonymous		0	0.06	1.17	3.7	13.02
*MESP2*	NM_001039958.1:c.829G>A	p.Gly277Ser	Missense	Spondylocostal dysostosis 2	0	0	0.19	-1.34	0.003

MAF: minor allele frequency, the maximal populational allele frequency of the variant obtained from 1000 genome database, gnomAD, and an in-house database; pLI, missense Z-score: obtained from the gnomAD database

To screen for potential pathogenic variants that were not covered by exome sequencing, we also performed whole genome sequencing on the proband. No reported pathogenic variants or cryptic splicing variants were identified. We also analyzed the regulatory elements of the candidate genes (i.e., *BRAF*, *FRAS1*, *ELF4*, and *MESP2*) from the pedigree analysis and did not find any potential deleterious variants.

Of the four co-segregating variants, the *BRAF* variant (NM_004333.6: c.362C > A) is predicted to be highly deleterious by Combined Annotation Dependent Depletion (CADD > 20) and highly conserved across various species (Fig. 1C). In addition, *BRAF* has a high probability of loss-of-function intolerance score (pLI = 1.0). Therefore, we prioritized the *BRAF* variant under the hypothesis of a dominant inheritance mode. The variant was confirmd by Sanger sequencing (Fig. 1D).

The *BRAF* gene encodes B-Raf protein, a serine/threonine protein kinase central to the RAS/MAPK signaling pathway. It is expressed in multiple organs, including the gonads and urinary tract. To date, only one variant in *BRAF* (c.16_40del) has been implicated in a sporadic case of midshaft hypospadias. But the gene-disease association was uncertain [12]. *BRAF* is an established causal gene for several autosomal dominant RASopathies, including Cardiofaciocutaneous syndrome (MIM 115150), Noonan syndrome (MIM 613706), and LEOPARD syndrome (MIM 613707). Common manifestations of *BRAF*-related syndromes include growth retardation, intellectual/motor developmental delay, congenital heart defects, craniofacial deformities, abnormal pigmentation of the skin, spare/curly hair, and cryptorchidism [13–15]. In the follow-up examination, we only observed trivial overlap between *BRAF*-related phenotypes and the clinical manifestation of the two patients (Table 1). The proband’s affected brother (III-2) has a mild constricted ear and a café-au-lait-like patch on the back. The proband’s affected uncle (II-4) has a low birth weight (1.75 kg) and curly hair. Therefore, we hypothesized that the *BRAF* variant might lead to hypospadias via a non-canonical mechanism. Therefore, we performed the subsequent functional assays to evaluate whether the variant altered MAPK signaling cascades and downstream gene expression.

### The *BRAF* c.362C > A variant results in p38 MAPK hyperphosphorylation

**Fig. 2:**
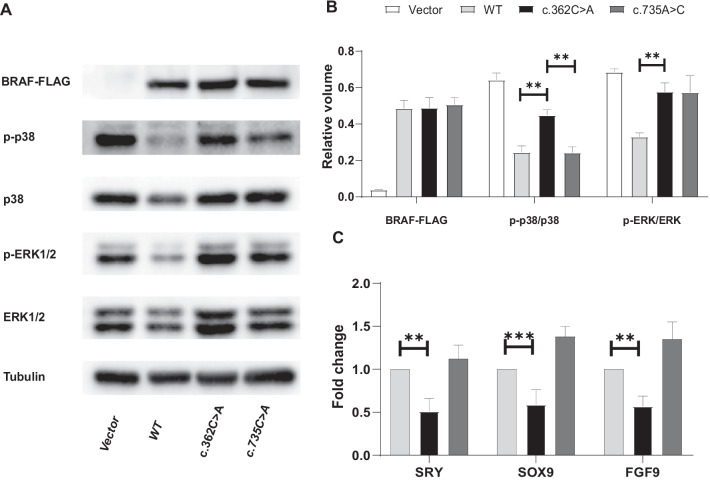
**A**, **B** Western blot analysis of total and phosphorylated MAPKs in cultured HEK293 cells overexpressing the BRAF mutant allele. Vector: transfected with the empty vector; WT: transfected with wildtype BRAF construct; c.362C > A: transfected with the novel *BRAF* variant (NM_004333.6: c.362C > A, p.Arg121Thr); 735A > C: transfected with the positive control variant (NM_004333.6:c.735A > C, p.Leu245Phe), a known pathogenic variant associated with LEOPARD syndrome 3. The proportion of phosphorylated p38 MAPK increased significantly in the c.362C > A group (44.80% vs 24.38%, p-value = 0.0016), while did not change significantly in the positive control group (24.24% vs 24.38%). The proportion of phosphorylated ERK MAPK increased significantly in both c.362C > A group (57.71% vs 32.85%, p-value = 0.001) and the positive control group (57.28% vs 32.85%, p-value = 0.013), but did not differ significantly between the two mutant groups(p-value = 0.963). **C** Quantitative PCR analysis of male-sex-differentiation-related genes. The expression of SRY (p-value = 0.002), SOX9(p-value = 0.0003), and FGF9 (p-value = 0.007) decreased by 50%, 42%, and 41% respectively in the c.362C > A (p.Arg121Thr) group. While in the positive control group, no reduction in expression of these gene was observed

We performed Western Blot on the lysate of cells overexpressing the wildtype *BRAF* or *BRAF* mutants. The mutants included the variant we identified (NM_004333.6:c.362C > A, p.Arg121Thr) and a positive control variant (NM_004333.6:c.735A > C, p.Leu245Phe) known to be associated with LEOPARD syndrome 3 [13]. The expression of BRAF protein was consistent between the wild-type and mutant cells (p-value = 0.61) (Fig. 2A, B). The proportion of phosphorylated ERK1/2 MAPK was increased in both mutants (p-value = 0.001 for c.362C > A; p-value = 0.013 for c.735A > C) but did not differ significantly between the two mutants (p-value = 0.946). Notably, the proportion of phosphorylated p38 MAPK increased significantly in the c.362C > A mutant (p-value = 0.0016) but not in the c.735A > C mutant (p-value = 0.963) (Fig. 2B), indicating that the c.362C > A variant had a distinct impact on p38 MAPK signaling.

Similarly, pathogenic *MAP3K1* variants associated with 46, XY sex reversal (MIM 613762) could cause hyperphosphorylation of ERK (downstream effector of BRAF) and p38 MAPKs by increased binding to MAP3K4 and RHOA. This hyperphosphorylation may account for the decreased expression of testicular-determining genes, which leads to testicular agenesis and the subsequent feminization of the male genitalia [27]. Therefore, the effect of the *BRAF* c.362C > A variant on p38 MAPK may account for the phenotypic disparity between the RASopathy phenotypes and the phenotypes of our patients.

### The *BRAF* c.362C > A variant is associated with a decreased *SRY* transcription

The hyperphosphorylation of ERK1/2 and p38 MAPKs caused by pathogenic *MAP3K1* variants was reported to decrease the expression of male-sex-differentiation-related genes [16]. Therefore, we examined the transcription level of *SRY*, *SOX9*, and *FGF9* genes in NT-2 cells overexpressing the wild-type or the two *BRAF* mutants (c.362C > A and c.735A > C). Compared to the cells overexpressing wild-type *BRAF*, *SRY* expression decreased by 50% in the c.362C > A group (p-value = 0.002) and increased by 6% in the c.735A > C group with a borderline significance (p-value = 0.071). A significant decrease in *SOX9* (42%, p-value = 0.0003) and *FGF9* (41%, p-value = 0.007) expression was also observed in the c.362 C > A group (Fig. 2C), but not in the c.735 A > C mutant group. During male sex differentiation, the *SRY* gene is the initiator, and the *SOX9* and *FGF9* genes are both crucial drivers. Decreased expression of these genes may lead to insufficient or delayed androgen production, thereby causing hypospadias. Our finding suggests that the *BRAF* c.362C > A variant can lead to decreased expression of testicular determining genes, consistent with the reported pathogenesis of hypospadias [16].

### Candidate variants in p38 MAPK-related genes in the sporadic hypospadias cohort

To search for more p38 MAPK-related genes that are potentially causal for hypospadias, we conducted pathway-based variant prioritization in the sporadic hypospadias cohort. Two variants in p38 MAPK-related genes, namely *TRIM67* p.R416T (NM_001004342.3:c.1247G > C) and *DAB2IP* p.G694D (NM_138709.2:c.2081G > A) (Table 4) were prioritized. Both variants are of maternal origin and have CADD scores higher than 20. No diagnostic variant was found in patients carrying these two candidate variants.

**Table 4:** Detailed information of candidate variants involved in MAPK signaling pathway

Patient ID	Hypospadias	Gene	Variant	Origin	MAF	MisZ	GERP + +	CADD
HSP2004P0199	Midshaft	*TRIM67*	NM_001004342.3:c.1247G>C	Maternal	0	2.79	5.61	22.9
HSP21034P0006	Midshaft	*DAB2IP*	NM_138709.2:c.2081G>A	Maternal	0	2.61	4.69	26.1

MAF: minor allele frequency, the maximal populational allele frequency of the variant obtained from 1000 genome database, gnomAD, and an in-house database; MisZ: missense Z-score obtained from the gnomAD database; GO BP: the biological process which the gene is involved in, obtained from the gene ontology database

*TRIM67* encodes the Tripartite Motif Containing 67 protein. It is a mediator of *MAPK11*, which encodes one of the p38 MAPKs. A recent study reported that *TRIM67* negatively regulates *MAPK11* expression [17]. A deleterious variant in this gene might affect the p38 signaling cascade. Further analysis is required to validate how the *TRIM67* p.R416T variant affects the p38 cascade.

*DAB2IP* is ubiquitously expressed and plays a diverse role in different MAPK signaling circuits. It negatively regulates RAS/MAPK signaling and thereby inhibits ERK1/2 phosphorylation; upregulates JUN and p38 MAPKs via activating ASK1 [18]. A deleterious variant in *DAB2IP* could potentially alter both the ERK1/2 cascade and the p38 cascade. The *DAB2IP* variant we prioritized is absent from public databases and has a CADD score of 26.1, suggesting its pathogenic potential. Nevertheless, its pathogenicity remains to be validated by functional assays.

## Discussion

In this study, we performed exome sequencing in a Chinese pedigree with three hypospadias patients and a sporadic cohort including 49 hypospadias patients. A co-segregating novel variant in the *BRAF* gene (NM_004333.6: c.362C > A) was identified from the pedigree. In vitro functional analysis conducted in cell lines overexpressing the mutated *BRAF* demonstrated hyperphosphorylation of p38 MAPK and decreased expression of *SRY*, *SOX9*, and *FGF9* genes. From the sporadic hypospadias cohort, we identified two rare deleterious variants in p38 MAPK-related genes, further highlighting the role of p38 MAPK-related genes in hypospadias.

**Fig. 3:**
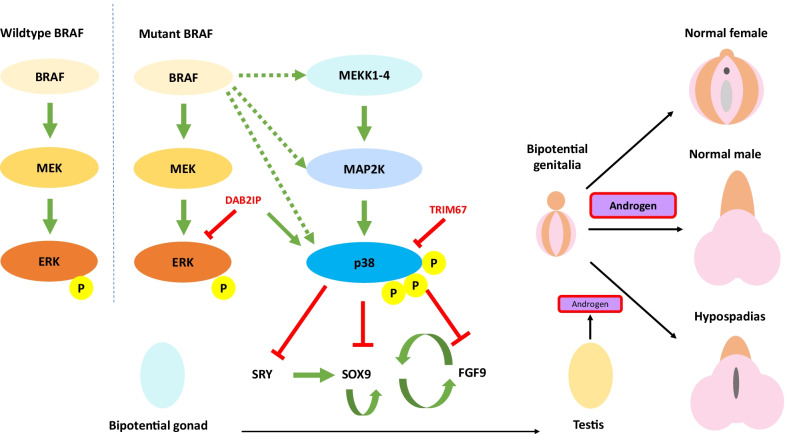
A schematic diagram demonstrating how the BRAF variant leads to hypospadias. Green bars indicate activation. Red bars indicate inhibition. Dash lines indicate potential interaction

In previous studies, pathogenic *BRAF* variants have been associated with a series of RASopathies. Phenotypes of *BRAF*-related RASopathies include intellectual instability, motor development delay, short stature, sparse/curly hair, pigmentation abnormality of skin, congenital heart defects, and craniofacial dysmorphism [13, 15]. Our patients, however, presented with only hypospadias but not the typical manifestations of *BRAF*-associated RASopathies. Variants causing *BRAF*-related RASopathies could increase the activation of ERK1/2 MAPK signaling [13]. In contrast, the variant we identified also increased the activation of p38 MAPK, while no significant change in p38 activation was observed for the RASopathy-related *BRAF* variant (Fig. 3). This distinct impact on p38 MAPK signaling might account for disparity between the phenotypes of our patients and *BRAF*-associated RASopathy phenotypes.

MAPK cascades are vital for male sex differentiation. Pathogenic *MAP3K1* variants that cause 46, XY sex reversal had increased binding to *RHOA*, *FRAT1*, and *MAP3K4* and upregulated ERK MAPK and p38 MAPK signaling [16, 19]. This increase in ERK1/2 and p38 MAPK signaling upregulated the expression of *FOXL2* and β-catenin, i.e., genes that drive female sex differentiation, while suppressed the expression of *SRY*, *SOX9*, and *FGF9*, i.e., genes that drive male sex differentiation [16]. Similarly, our findings also suggest that p38 overactivation caused by the *BRAF* variant could downregulate the expression of the *SRY* gene and thereby inhibit male sex differentiation.

Besides the *BRAF* variant, we also identified a co-segregating variant in *FRAS1*, a gene known to be associated with hypospadias. The known inheritance patterns of *FRAS1*-related disorders include recessive and compound heterozygous inheritance. Both exome sequencing and whole genome sequencing were performed on the proband, but neither yielded any additional candidate variant in *FRAS1* or its regulatory elements. Nevertheless, we cannot rule out the potential contribution of the *FRAS1* variant to the disease pathogenesis via a multi-genic mechanism.

Another limitation of our study is the modest relevance of the two variants identified in the sporadic cohort. One the one hand, the sample size of the sporadic cohort is limited. On the other hand, no functional assessment was carried out to test the effect of the two variants on p38 MAPK signaling. Besides, although no diagnostic variant was identified, the contribution of other rare deleterious variants cannot be ruled out either. Future studies could validate the relevance of these variants in larger cohorts and test their molecular impact with functional assays.

## Conclusion

In this study, we identified and validated a novel *BRAF* variant (NM_004333.6: c.362C > A, p.Thr121Lys) causal for hypospadias. Besides, we identified two candidate variants involved in p38 MAPK signaling that may be associated with hypospadias. Our study expands the phenotypic spectrum of variants involved in p38 MAPK signaling toward the involvement of hypospadias.

## Materials and methods

### Participants and clinical evaluation

In this study, patients affected by isolated hypospadias with potential genetic predispositions were included and subjected to exome sequencing. From October 2016 to November 2020, children admitted to the Department of Urology of Beijing Children's Hospital for surgical repair of hypospadias were evaluated to determine eligibility. All the patients underwent routine examinations to rule out 46, XY Disorders of Sexual Development. Gestational exposure to potential teratogens (diethylstilbestrol, pesticides, herbal medicine) and gestational complications (assisted reproductive technology, infection in the first trimester, preeclampsia, gestational diabetes, etc.) were investigated to exclude hypospadias caused by environmental or maternal factors [3, 6]. We preliminarily analyzed the exome sequencing data of the patients and excluded those carrying known pathogenic variants. In total, we enrolled a pedigree with three hypospadias patients in two generations (Fig. 1A) and a sporadic cohort including 49 hypospadias patients. For the sporadic cohort, we also enrolled the parents of 29 patients and the healthy brothers of the other 20 patients to facilitate the subsequent genetic analysis.

To compare the phenotypes of our patients with *BRAF*-related syndromes (Cardiofaciocutaneous syndrome MIM: 115150, LEOPARD syndrome 3 MIM: 613707, Noonan syndrome 7 MIM: 613706) [13, 15], a follow-up systemic evaluation was performed on each patient in the pedigree. We inspected the patients for cutaneous abnormalities (such as multiple lentigines, hyperpigmentation, spare hair, curly hair, ichthyosis, and eyebrow hypoplasia) and craniofacial deformities (such as short-neck, auricular deformity, macrocephaly/microcephaly, hypertelorism, epicanthus, and depressed nasal bridge). Echocardiogram and urinary tract ultrasound were performed to identify potential heart defects and urogenital anomalies.

### Exome sequencing

We collected blood samples from the participants and extracted DNA from peripheral blood lymphocytes with standard methods. For the disease pedigree, we performed exome sequencing on the patients and the available family members, seven subjects in total (Fig. 1A). For the sporadic cohort, we sequenced 49 hypospadias patients. For 29 of them, exome sequencing was also performed in their parents. For the remaining 20 patients, their healthy brothers were sequenced. DNA samples were prepared in Illumina libraries and then underwent whole-exome capture with the SureSelectXT Human All Exon V6. The subsequent sequencing was performed on the NovaSeq S4 PE150 platform.

### Variant calling and preliminary filtration

All variants were called and annotated using an in-house developed Peking Union Medical College Hospital Pipeline (PUMP) [20–22]. Human reference genome hg19 was used for all analyses. We retained variants that pass the quality control threshold (genotype quality > 20, read depth > 10, variant allele frequency > 30). The populational frequency of each quality-control-passed variant was obtained from the public population databases, including the 1000 genome project, the Exome Sequencing Project [23], the Genome Aggregation Database (gnomAD) [24], and the in-house database of DISCO (Deciphering disorders Involving Scoliosis and COmorbidities, http://discostudy.org/, ≈ 8000 exomes/genomes) study. Rare variants (minor allele frequency < 0.001) were retained for further filtering. From these rare variants, we included the protein-altering or splice-region variants for subsequent analysis.

### Pedigree data analysis

For the pedigree data analysis, we assumed a sex-limited autosomal/X-linked dominant mode of inheritance. Therefore, we included the variants that are present in the patients (III-1, III-2, and II-4) and the proband’s mother but absent in the proband’s father (II-1) (see Fig. 1A). For the remaining variants, we selected the variants with CADD scores (v1.2) above 20.

### Whole genome sequencing

We performed whole genome sequencing on the proband of the hypospadias pedigree to screen for potential pathogenic variants that were not covered by exome sequencing, including cryptic splice sites, copy number variants (CNVs), and structural variants (SVs). The peripheral DNA was extracted following standard protocols. Sequencing libraries were prepared using the KAPA Hyper Prep kit (KAPA Biosystems, Kusatsu, Japan) with an optimized manufacturer’s protocol. We performed multiplex sequencing using an Illumina HiSeq X-Ten sequencer (Illumina, San Diego, CA, USA). Variants were called and annotated using the PUMP [20–22]. We excluded the variants that did not pass our quality-control filters (genotype quality > 20, read depth > 10, variant allele frequency > 30). We first looked for known pathogenic variants reported in the ClinVar database(https://www.ncbi.nlm.nih.gov/clinvar/, version 2022-01-27) or the Human Gene Mutation Database (HGMD) [25, 26]. We predicted the impact of intronic variants on splicing using the SpliceAI software [27]. Regulatory element annotation for each variant was retrieved from the Regulatory Elements Database [28]. CNVs were called using the CNVnator v0.4.1 software using the default parameters [29]. SVs were called using the LUMPY v0.3.1 software following standard procedures [30]. CNVs and SVs were annotated using the AnnotSV 3.0 webserver [30, 31]. We looked for CNVs/SVs that overlap with known pathogenic variants in the DECIPHER database v11.9 [32]. We also looked for CNVs/SVs that overlap with the four candidate genes (*BRAF*, *FRAS1*, *MESP2*, and *ELF4*) or their regulatory elements.

### Cohort data analysis

To further explore the role of MAPK signaling in hypospadias, we searched for variants in MAPK signaling-related genes. Genes involved in MAP kinase activity (GO: 0004707), MAPK cascade (GO: 0000165), regulation of MAPK cascade (GO: 0043408), and regulation of p38 MAPK cascade (GO:1900744) were selected for further analysis. For the patients whose parents were sequenced, we excluded paternally inherited variants. For patients whose healthy brothers were sequenced, we excluded variants that are present in the healthy siblings. For patients who carried candidate variants in the above pathways, we reviewed their individual exome data for other potentially causal variants according to the American College of Medical Genetics and Genomics and the Association for Molecular Pathology (ACMG/AMP) guideline for germline variant classification [33].

### Variant confirmation

The *BRAF* variant we identified is in a low-complexity region, which might be prone to sequencing errors. Therefore, we performed Sanger sequencing to confirm the variant. Primers used in Sanger sequencing were designed and validated using standard methods. The primers we used are as follows: forward primer: 5’-CAGGACAAAGTCCGGATTGA-3’; reverse primer: 5’-GGATGCCTCTATTTGCATGACC-3’.

### Plasmid construct and cell line construct

To analyze the functional consequences of the candidate *BRAF* variant, we constructed a pCMV4-FLAG-BRAF plasmid using a pEGFP-BRAF plasmid and a pCMV4-FLAG vector (purchased from Suzhou Bio-research Innovation Center, Chinese Academy of Sciences). A FLAG-tag was added to the N-terminus of BRAF protein to facilitate detection. A BRAF variant (NM_004333.6:c.735A > C, p.Leu245Phe) known to cause LEOPARD syndrome 3 was used as a positive control [13]. The candidate variant (NM_004333.6:c.362C > A, p. Thr121Lys) and the positive control variant were introduced by site-directed mutagenesis (KOD-Plus-Mutagenesis Kit, TOYOBO, Shanghai, China) into the pCMV4-FLAG-BRAF plasmids.

HEK 293 T cells were cultured using DMEM medium supplied with 10% fetal bovine serum following standard protocol. NT-2 cells were cultured using DMEM high glucose supplied with 10% fetal bovine serum and streptomycin (10^4^ μg/mL) in a 37℃ container at 5% CO_2_. We used 0.25% trypsin to detach the cells when they reach 90% confluency and split 1:3 with fresh growth media. Both cell lines were transfected with either the null vector, the wild-type *BRAF* construct, or one of the *BRAF* mutant constructs following the lipofectamine 3000 protocol.

### Western blot

HEK293 cells overexpressing the mutant or the wild-type *BRAF* were used to evaluate the effects on the MAPK signaling cascade exerted by the variants. 24 h after transfection, cells were switched to serum-deprived medium for 12 h and lysed in a standard lysis buffer supplemented with phosphatase inhibitor. Cell lysates were run on 10% SDS-PAGE, transferred onto nitrocellulose membrane, blocked with bovine serum albumin, and examined for the basal phosphorylation levels of ERK and p38 MAPKs using the following antibodies: p44/42 MAPK (Erk1/2) rabbit antibody, Phospho-p44/42 MAPK (T202/Y204) rabbit antibody, p38 MAPK (D13E1) XP® Rabbit mAb, and Phospho-p38 MAPK (Thr180/Tyr182) (D3F9) XP® Rabbit mAb (Cell Signaling Technology). The expression level of BRAF protein was examined using a rabbit anti-FLAG polyclonal antibody (Applygen, Beijing). GAPDH was loaded as the internal control.

### Quantitative PCR

The NTERA-2 (NT-2) cell line is a human embryonic carcinoma cell line derived from testicular teratoma. NT-2 expresses testicular-determining genes, thus an appropriate in vitro tool for studying male sex differentiation and development [34]. We used NT-2 cells overexpressing the mutated/wild-type BRAF to evaluate the effect of the *BRAF* variant on the expression of male-differentiation-related genes. 24 h after transfection, cells were lysed and total RNA was isolated. Quantitative PCR experiments were performed using the TaqMan gene expression master mix. β-actin was amplified as an internal control. The primers used for the assay were obtained from the PrimerBank database (https://pga.mgh.harvard.edu/primerbank/) and validated using the UCSC in-silico PCR tool (https://genome.ucsc.edu/cgi-bin/hgPcr).

The western blot and Quantitative PCR assays were repeated for three times. Difference between groups were compared using the Student’s t test.

## Data Availability

The datasets used and/or analyzed during the current study are available from the corresponding author on reasonable request.
